# Epidemiology and outcomes of critically ill patients in the emergency department of a tertiary teaching hospital in Rwanda

**DOI:** 10.1186/s12245-024-00736-9

**Published:** 2024-11-05

**Authors:** Laurent Gamy Kamunga B., Courtney J. Bearnot, Kyle D. Martin, Doris L. Uwamahoro, Giles N. Cattermole

**Affiliations:** 1 Department of Anesthesia, Critical Care, and Emergency Medicine, College of Medicine and Health Sciences, University of Rwanda, Kigali, Rwanda; 2 Accident and Emergency Department, King Faisal Hospital Rwanda, Kigali, Rwanda; 3 Department of Emergency Medicine, Brigham and Women’s Hospital, Boston, MA USA; 4 Department of Emergency Medicine, Brown University Warren Alpert Medical School, 222 Richmond Street, Providence, RI 02912 USA; 5 King’s College Hospital NHS Foundation Trust, London, Great Britain

**Keywords:** Emergency care, LMIC, Critical care, Resuscitation, Rwanda

## Abstract

**Background:**

The introduction of Emergency Medicine in Rwanda in 2015 has been associated with a mortality reduction in patients presenting to Kigali University Teaching Hospital (KUTH). In the context of increasing numbers of critically ill patients presenting to Emergency Departments (ED) globally, the aim of this study was to describe the characteristics of critically ill patients, the critical care interventions performed, and the outcomes of critically ill patients presenting to the KUTH ED with the goal of informing future research into the root causes of mortality of critically ill ED patients and of identifying high yield topics for didactic and procedural training.

**Methods:**

A descriptive observational prospective cohort pilot study analyzed all patients ≥15 years who presented to KUTH between April and June 2022 with modified South African Triage Scores of Red with alarm, Red without alarm, and Orange.

**Results:**

Of 320 patients, 66.9% were male and median age was 40 years. Patients were triaged as Orange (65.3%), Red without alarm (22.8%), and Red with alarm (11.9%). Presentations were categorized as: medical emergencies (48.0%), traumatic injury (44.5%), and surgical emergencies (7.6%). Median length of stay was 31 h (IQR 28, 56) and boarding was 23 h (IQR 8, 48). Overall mortality was 12.2% and highest among medical emergencies (16.5%, *p* = 0.048) and increased significantly with triage color: Red with alarm (47.4%), Red without alarm (16.4%), and Orange (4.3%, *p* < 0.0001). Cardiopulmonary resuscitation (CPR) (10.3%), endotracheal intubation (8.8%), and vasopressor administration (3.1%) were the most frequent critical interventions performed. Survival after cardiac arrest was 9.1% and 32.1% after intubation. Mortality was associated with the following interventions: CPR, intubation, and use of vasopressors (*p* < 0.05).

**Conclusions:**

This pilot study identified the most common critical care interventions performed and a high mortality among patients who required these interventions in the ED of a tertiary teaching hospital in Rwanda. These findings will inform didactics and procedural training for emergency care providers. Future research should focus on the root causes of mortality in these specific patient populations and identify areas of system strengthening to reduce mortality.

**Supplementary Information:**

The online version contains supplementary material available at 10.1186/s12245-024-00736-9.

## Introduction

Where emergency care is available, critical care typically starts in the Emergency Department (ED). Globally, there is an increasing number of critically ill patients being treated in EDs. Between 2006 and 2014 in the United States, the number of critically ill patients cared for in the ED increased by approximately 80%, including a 16% increase in the number of intubated patients [1]. Prior research has shown that in-hospital mortality increases with decreasing gross national income [2] and that mortality is higher in low- and middle-income countries (LMIC) Intensive Care Units (ICU) compared to high-income countries (HIC) [3]. This has been attributed to multiple factors including high staffing ratios, poor access to medications and equipment, limited ICU bed availability, and lack of prioritization of emergency and critical care [4–6]. A Canadian study by Green et al., showed that the majority of patients admitted to the ICU received at least one critical care procedure in ED, such as endotracheal intubation, central venous catheter (CVC) insertion, arterial catheter insertion, and chest tube insertion, 64%, 17.9%, 14.1% and 4.5% respectively [7]. 

In Rwanda, a four year Emergency Medicine (EM) residency training program at Kigali University Teaching Hospital (KUTH) was introduced in 2015 and has been associated with a 43% reduction in overall hospital mortality likelihood [8]. However, data regarding critical care provided and subsequent patient outcomes in LMIC EDs including Rwanda are sparse. Patients triaged as critically ill have a higher risk of mortality though no data on critical care provided to these patients were reported [9]. Mbanjumucyo et al. showed that despite a similar first attempt intubation success rate at KUTH, intubated patients have a higher mortality rate compared to intubated patients in HIC [5]. In Bhutan, the most common critical care interventions performed in the ED were intubation (28.3%), CVC insertion (20.7%), and hemodialysis (16.6%) and mortality of critically ill patients was 38.0% [10]. The frequency of critical care interventions at KUTH and the outcomes of patients receiving these interventions has yet to be reported.

The aim of this pilot study was to describe the characteristics of critically ill patients, the critical care interventions performed, and the outcomes of critically ill patients presenting to the KUTH ED with the goal of informing future research into the root causes of mortality of critically ill ED patients and of identifying high yield topics for didactic and procedural training.

## Methods

### Population and location characteristics

We performed an observational prospective cohort pilot study from April to June 2022 of critically ill patients who presented to the KUTH ED, the main public referral and university teaching hospital in Rwanda. KUTH houses Rwanda’s sole EM, critical care, and anesthesia training programs. During our study, KUTH had approximately 500 inpatients beds, including 7 ICU beds, and 24 ED beds as well as 5 beds for isolation. KUTH has since increased its capacity to 11 ICU beds. The hospital has an annual volume of approximately 20,000 ED visits and 300 ICU admissions. Triage at the KUTH ED is based on the modified South African Triage Score (mSATS) which has been validated at KUTH ED [9]. mSATS is a modified version of the South African Triage Score (SATS) which was developed for the South African context and has since been adapted to multiple African counties [11–13]. In Rwanda, mSATS is used to triage patients into one of five colors – Green, Yellow, Orange, Red without alarm, and Red with alarm – based on signs of emergent distress, vitals, and presentation (Fig. S1).

For this study, critically ill patients were defined as those who were triaged as Red with alarm, Red without alarm, or Orange. All patients aged 15 years and older who were triaged as critically ill during the study period were eligible for enrollment. Patients triaged as Yellow or Green and those under the age of 15 were excluded.

### Data collection

Data including age, sex, triage category, mode of arrival, time of admission or discharge, time of ED departure, intervention(s) provided, and outcome were recorded onto a written standardized data collection form that was placed into the patient’s chart upon receiving consent. Clinical providers completed the data collection form. The form was collected from the patient’s chart and reviewed for completion upon patient disposition from the ED. Each patient was assigned a unique patient identifier. To maintain patient confidentiality, this unique identifier was linked to their hospital identification number and kept in a separate secured document. All data were deidentified prior to entry in an electronic database. ED length of stay (LOS) was measured as the time of registration to time of departure from the ED. Boarding time was defined, per the American College of Emergency Physicians policy, as from the time of decision to admit a patient to an inpatient service, to time of transfer to an inpatient unit [14]. 

### Statistical analysis

Data were analyzed using SAS (SAS Institute Inc. Cary, NC). Descriptive statistics including median and interquartile range or count and percentages were calculated for cohort characteristics and frequency of interventions. Pearson’s chi-square test or Fisher’s exact test were used to calculate p-values for associations with frequency of critical care interventions performed and associations with mortality. A p-value of 0.05 was considered statistically significant.

### Ethical approval and consent

We obtained ethical approval from the University of Rwanda IRB committee (No 178/CMHS IRB/2022) and KUTH Research Committee (EC/CHUK/044/2022). Eligible patients (or their caretaker) were consented prior to enrollment. All consent and data collection forms were locked in a well-protected cupboard accessible only by the principal investigator.

Any patient who met inclusion criteria was approached to be enrolled in this study by the physician caring for the patient at the time or by a physician member of the research team once the patient was stabilized. If the patient was unable to consent due to their clinical condition, their caregiver was approached. Consent for enrollment occurred prospectively as patients presented to the ED. Of those patients approached, 100% consented to be included in the study. All physicians who staff the ED in this setting were educated on the study and were able to enroll patients such that enrollment was possible 24 h a day.

## Results

A total of 320 patients were enrolled. No patients declined participation in the study. The median age was 40 years (IQR 28, 56) and 66.9% were male (Table 1). The most common mode of arrival was self-presentation (42.0%) and transfer from district hospitals (41.3%), followed by 17.2% by Emergency Medical Services ambulance. Patients were categorized as Orange (65.3%), Red without alarm (22.8%), and Red with Alarm (11.9%). Nearly half (48.0%) presented for medical complaints, 44.1% due to physical trauma, and 7.5% due to non-traumatic surgical complaints.

Half of all patients were admitted to the medical wards (50.6%), 24.7% were discharged, 7.8% were admitted to the operating room (OR), 3.8% were admitted to the ICU, and 0.9% were transferred to another hospital. Median LOS was 31 h (IQR 14, 61) and median boarding was 23 h (IQR 8, 48). Overall mortality in the ED was 12.2%. Of the 39 patients who did not survive, 11 patients (28.2%) had not yet been admitted and 28 deaths (71.8%) occurred while boarding. Of these 28 patients, 17 (60.7%) were boarding for an ICU bed and 11 (39.2%) were boarding for a ward bed.

**Table 1:** Patient demographics and case characteristics

	*N* = 320	%
Age, median (IQR)	40	(28, 56)
**Sex**		
Male	214	66.9
Female	106	30.0
**Triage Category**		
Orange	209	65.3
Red without alarm	73	22.8
Red with alarm	38	11.9
**Mode of arrival**		
Self-presented	133	41.6
Transfer from district hospital	132	41.3
Ambulance	55	17.2
**Chief complaint**		
Trauma	141	44.5
Medical	152	48.0
Surgical	24	7.6
**Length of Stay (Hours)**		
Median (IQR)	31	(14, 61)
**Disposition**		
Discharge home	79	24.7
Admission to ward	162	50.6
Admission to ICU	12	3.8
Admission to operating room	25	7.8
Transfer to another hospital	3	0.9
Death	39	12.2

*Percentages may not sum to 100% due to rounding

Of the 320 patients, 74 (23.1%) had one or more critical care interventions performed: 33 (10.3%) received cardiopulmonary resuscitation (CPR), 28 (8.8%) were intubated and mechanically ventilated, 10 (3.1%) required vasopressor support, 9 (2.8%) received a thoracentesis or tube thoracostomy, 6 (1.9%) had a CVC placed, 4 (1.4%) received a pericardiocentesis, and 3 (0.9%) received non-invasive positive pressure ventilation (NIPPV) (Table 2). Of the six patients who had a CVC placed, one received vasopressors. Approximately one-third of patients who received an intervention required two, three, or four interventions – 17 (22.3%), 4 (5.4%), and 1 (1.4%) patient, respectively. Intubation and CPR were the most frequently combined and were performed on 13 (17.6%) patients.

There were no significant differences in critical care interventions provided to males and females (*p* = 0.322). Those transferred from a district hospital were more likely to require CPR (14.4%) compared to those presenting via ambulance and self-presenting (12.7% and 5.3% respectively, *p* = 0.041). Patients presenting with surgical and medical complaints were more likely to require any intervention compared to those presenting with traumatic injury (29.2%, 29.1%, 15.6% respectively, *p* = 0.018). Surgical emergencies required vasopressors (12.5%, *p* = 0.007) and NIPPV (8.3%, *p* < 0.001) more frequently than either medical (4.0% and 0.7%, respectively) or trauma patients (0.7% and 0.0%, respectively). Critical care interventions were performed on more patients triaged as Red with alarm (57.9%) compared to patients triaged as Red without alarm (34.3%), and Orange (12.9%, *p* < 0.001). In those triaged as Red with alarm, the interventions that were performed most frequently were: CPR (42.1%, *p* < 0.001) and intubation (34.2%, *p* < 0.001). Thoracenteses were performed more frequently in those triaged as Red without alarm (8.2%) compared to Red with alarm (2.6%) and Orange patients (1.0%, *p* = 0.005).

**Table 2:** Characteristics of critical care interventions performed

Intervention		Mode of arrival, *n* (%)					Chief complaint, *n* (%)					Triage category, *n* (%)			
	Overall *N* = 320	Self-presented	Ambulance	Transfer	*p*-value		Trauma	Surgical	Medical	*p*-value		Orange	Red without alarm	Red with alarm	*p*-value
**Any intervention**	74 (23.1)	23 (17.3)	12 (21.8)	39 (29.6)	0.059		22 (15.6)	7 (29.2)	44 (29.1)	0.018		27 (12.9)	25 (34.3)	22 (57.9)	< 0.001
**CPR**	33 (10.3)	7 (5.3)	7 (12.7)	19 (14.4)	0.041		10 (7.1)	3 (12.5)	19 (12.6)	0.276		7 (3.4)	10 (13.7)	16 (42.1)	< 0.001
**Intubation**	28 (8.8)	7 (5.3)	5 (9.1)	16 (12.2)	0.141		11 (7.8)	1 (4.2)	15 (9.9)	0.588		6 (2.9)	9 (12.3)	13 (34.2)	< 0.001
**Vasopressor**	10 (3.1)	4 (3.0)	1 (1.8)	5 (3.8)	0.776		1 (0.7)	3 (12.5)	6 (4.0)	0.007		4 (1.9)	4 (5.5)	2 (5.3)	0.232
**Tube Thoracostomy**	9 (2.8)	4 (3.0	1 (1.8)	4 (3.0)	0.887		4 (2.8)	0 (0.0)	5 (3.3)	0.663		6 (2.9)	2 (2.7)	1 (2.6)	0.996
**Thoracentesis**	9 (2.8)	2 (1.5)	2 (3.6)	5 (3.8)	0.489		1 (0.7)	1 (4.2)	7 (4.6)	0.121		2 (1.0)	6 (8.2)	1 (2.6)	0.005
**Central venous access**	6 (1.9)	2 (1.5)	0 (0.0)	4 (3.0)	0.349		1 (0.7)	1 (4.2)	4 (2.7)	0.335		3 (1.4)	2 (2.7)	1 (2.6)	0.728
**Pericardiocentesis**	4 (1.3)	3 (2.3)	0 (0.0)	1 (0.8)	0.360		2 (1.4)	0 (0.0)	2 (1.3)	0.845		4 (1.9)	0 (0.0)	0 (0.0)	0.341
**Non-invasive ventilation**	3 (0.9)	1 (0.8)	0 (0.0)	2 (1.5)	0.593		0 (0.0)	2 (8.3)	1 (0.7)	< 0.001		2 (1.0)	1 (1.4)	0 (0.0)	0.778

Mortality was associated with triage category (Table 3). Of those deceased, 47.4% were triaged as Red with alarm, 16.4% as Red without alarm, and 4.3% as Orange (*p* < 0.0001) and were more likely to have a medical chief complaint (65.8%) than a trauma (25.6%) or surgical chief complaint (7.9%, *p* = 0.048). Among critical care interventions, those deceased were more likely to have undergone intubation, vasopressor use, and CPR (*p* < 0.001) compared to those who survived. However, tube thoracostomy was associated with survival (*p* = 0.049). There was no significant association between sex, mode of arrival, or length of stay and mortality. Median LOS was similar between groups (31 h vs. 29 h, *p* = 0.921). Median boarding time was shorter for those who survived (22 h vs. 31 h); however, this was not significant (*p* = 0.316).

**Table 3:** Characteristics associated with overall survival, (*N* = 320)

	Survived, *n* (%)	Deceased, *n* (%)	*p*-value
Age, median (IQR)	39 (29, 56)	41 (26, 60)	0.884
**Sex**			
Male	187 (66.6)	27 (69.2)	0.739
Female	94 (33.5)	12 (30.8)	
**Triage color**			
Orange	200 (95.7)	9 (4.3)	< 0.0001
Red without alarm	61 (83.6)	12 (16.4)	
Red with alarm	20 (52.6)	18 (47.4)	
**Arrival to ED**			
Self-attended	123 (43.8)	10 (25.6)	0.074
Transfer from District Hospital	110 (38.2)	22 (56.4)	
Brought by SAMU	48 (17.1)	7 (18.0)	
**Cause**			
Trauma	131 (47.0)	10 (26.3)	0.048
Medical	27 (45.5)	25 (65.8)	
Surgical	21 (7.5)	3 (7.9)	
**Interventions**			
Any intervention	37 (13.2)	37 (94.9)	< 0.0001
Intubation	9 (3.2)	19 (48.7)	< 0.0001
Non-invasive ventilation	2 (0.7)	1 (2.6)	0.324
Central venous access	5 (1.8)	1 (2.6)	0.545
Vasopressor	4 (1.4)	6 (15.4)	< 0.001
Tube thoracostomy	6 (2.1)	3 (7.7)	0.049
CPR	3 (1.1)	30 (76.9)	< 0.0001
Pericardiocentesis	4 (1.4)	0 (0.0)	1.000
Thoracentesis	7 (2.5)	2 (5.1)	0.302
**Length of stay and boarding duration**			
Median LOS (hours), (IQR)	31 (14, 59)	29 (9, 74)	0.921
Median boarding (hours), (IQR), *N* = 229	22 (8, 42)	31 (9, 59)	0.316
Median boarding > 24 h, *N* = 229	93 (46.3)	16 (57.1)	0.280

**Fig. 1:**
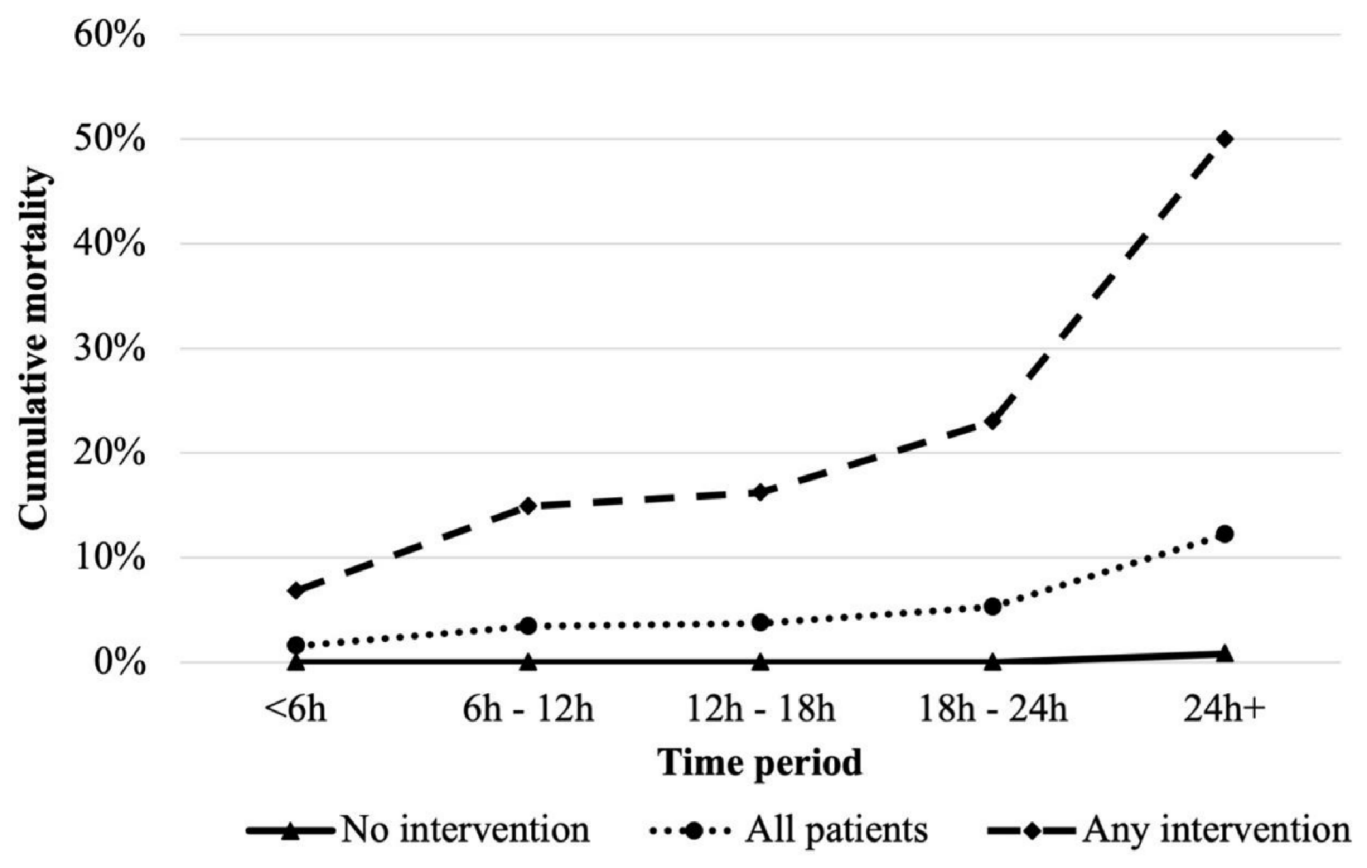
Cumulative mortality of patients by emergency department length of stay

Figure 1 shows cumulative mortality by ED LOS including subgroups of all patients, patients who received no intervention, and patients who received at least one intervention. The cumulative mortality for each group is shown in 6-hour intervals and after 24 h. Mortality increased drastically after 24 h for all patients, especially those who required critical care interventions.

## Discussion

This study describes the characteristics of critically ill patients, the critical care interventions performed, and the outcomes of 320 critically ill patients in the ED at a tertiary teaching hospital in Rwanda. Our cohort characteristics including age distribution, sex proportions, and disposition were similar to prior studies from the KUTH ED [9, 15]. Nearly a quarter of critically ill patients required a critical care intervention in the ED with CPR, intubation, and vasopressor administration being the most common. Less than 10% of patients who received CPR survived. Given the high mortality associated with critical care interventions especially CPR, new research should focus on identifying the root causes of mortality in this patient population. Additionally, didactics and procedural training on high-quality CPR, post-return of spontaneous circulation (ROSC) management, and ventilation management may be high yield.

Sparse data exist on CPR outcomes in LMIC. In HIC, in-hospital cardiac arrest mortality is decreasing and is associated with higher survival compared to out-of-hospital cardiac arrest [16]. Between 2000 and 2021 ROSC was achieved in 66.9% cases of in-hospital cardiac arrest cases in the United States and improved from 53.1 to 73.7% between 2000 and 2005 and 2016–2021. In the same cohort, 22.6% survived to hospital discharge between 2000 and 2021 [17]. Two systematic reviews of out-of-hospital cardiac arrest in 26 and 67 countries did not include data from the African region [18, 19]. Our pilot study was not designed to differentiate between out-of-hospital cardiac arrest with care initiated in the field and continued upon ED arrival and in-hospital cardiac arrest. However, future research aiming to understand the root cause of cardiopulmonary arrest and associated operational processes may better elucidate areas for improvement. For example, investigating if the primary cause was cardiac or respiratory, how quickly life support medications are available at the bedside, and the timing of respiratory support such as the availability of intubation equipment and ventilators would be important next steps.

Currently, many of the recommendations for Advanced Cardiac Life Support (ACLS) and post-resuscitation care such as targeted temperature management may not be possible in Rwanda given the lack of resources. A review of global pediatric advanced life support (PALS) interventions suggested that the resuscitation protocols used in HICs may not be clinically appropriate and specialized equipment and medications not available in LMIC [20]. Furthermore, a cross-sectional analysis of EM physicians from 23 African countries showed that approximately one-quarter did not have access to ACLS courses with East and West African regions reporting less access than North and South African regions [21]. A recent consensus statement highlighted the importance of context-specific resuscitation research and the development locally appropriate guidelines to improve outcomes of cardiac arrest in LMIC [22]. 

One barrier common to many critical care interventions is lack of equipment. KUTH ED is currently allocated 3 ventilators. As such, they are frequently used for intubated admitted patients who are waiting for an available ICU bed and are infrequently available for BIPAP and CPAP. This is likely the reason only 1.0% of patients received NIPPV. Similarly, a 2019 study of 64 patients from KUTH reported CVC placement in 11% of patients who required vasopressors [23]. In our cohort, 10% of patients who required vasopressors had a CVC placed. Our study did not differentiate the indication for CVC placement - vasopressor use or hemodialysis. While CVC placement is the standard of care for patients requiring vasopressors in HICs, barriers including availability of equipment and expense exist in LMIC [24]. 

ED LOS is increasing globally. Significant research in HICs has been dedicated to the association between ED LOS and mortality though few studies from LMIC exist. A 2022 systematic review of 50 papers and meta-analysis of 33 papers found that an ED LOS > 24 h and < 3 h was associated with mortality among patients admitted to the ICU and non-ICU patients, respectively [25]. However, none of these reviews included studies from African countries. This study showed a median LOS of 31 h and boarding of 23 h which is higher than studies in Bhutan (18 h) and Nepal (6 h) [10, 26]. The Bhutan study noted a crude OR of mortality of 1.52 for patients with ED LOS > 6 h though this was not significant (*p* = 0.082). Additionally, of two Ethiopian studies, one reported a ED LOS greater than 24 h in 91.5% of all ED patients but a median of 13.5 h for patients admitted to the ICU [27, 28]. Lastly, Barthelemy et al. reported worse outcomes for patients with traumatic brain injury in Cambodia when admission was delayed [29]. This study showed a trend towards longer boarding time for those who did not survive but this trend was not significant. This could potentially be due to a small sample size. In our cohort, median boarding time was 9 h shorter in those who survived but this difference did not reach significance. Cumulative mortality increased with ED LOS especially among patients who required critical care interventions. We hypothesize that lack of inpatient beds, especially in the ICU is the main contributing factor to boarding. However, further research evaluating the factors contributing to delay in admission and transport to the appropriate unit is needed.

### Limitations

Our study has several limitations. It is crucial to note that this pilot study was not designed to establish causation. The absence of intervention time data prevents the evaluation of intervention success although anecdotally, most interventions that a patient undergoes occur shortly after arrival and subsequent mortality occurs in the interim while the patient is awaiting a bed in the hospital. For example, it may be that patients represented in Fig. 1 were initially stabilized with a critical intervention but succumbed to their illness hours later while boarding in the ED. Nevertheless, this temporal ambiguity underscores the need for further investigations incorporating detailed temporal data to enable a more nuanced and accurate assessment of the impact of critical care interventions on patient outcomes.

Additionally, our data were obtained from a single study site which is a tertiary teaching hospital and relatively well-equipped compared to other hospitals in the country. Therefore, these results are unlikely to be generalizable to the district hospitals in Rwanda. In the future, this study can be extended to other tertiary hospitals in Rwanda which could elucidate systemic factors influencing the outcomes of critically ill patients. Also, in this pilot study, we did not collect data on the patient’s underlying comorbidities or final diagnosis which would likely provide more clarity on associations with mortality. Furthermore, this study was performed in the period of three months which could result in selection bias. The seasonality of medical, surgical, and traumatic conditions has yet to be documented in Rwanda but may vary throughout the year. Lastly, as has been demonstrated previously, increasing severity of triage category was significantly associated with mortality [9, 30]. Mortality in this cohort was higher than previously reported by Uwamahoro et al. [9] Their research however, showed that mSATS did not maintain its predictive power when applied to trauma patients. The difference in mortality rates by triage category could represent improvement in the triage of critically ill patients at KUTH. However, while scale-based triage protocols guide healthcare providers when assessing expected level of care, the accuracy is strongly influenced by the experience of the triaging provider [31]. 

## Conclusion

This pilot study identified a high mortality rate among patients who required critical care interventions in the ED of a tertiary teaching hospital in Rwanda and revealed prolonged boarding times in the ED. The frequency of the interventions performed will inform areas of focus for didactics and procedural training as well as future research. As patients requiring critical care in the ED increase, health systems and healthcare providers must be prepared to meet the needs of these patients. Based on our findings, it is crucial to increase in critical care training for emergency care providers, increase assess to equipment including ventilators and CVC and strengthen health systems such that they can safely accommodate more intensive care inpatient beds in order to improve ED flow.

## Electronic supplementary material

Below is the link to the electronic supplementary material.

Supplementary Material 1

## Data Availability

The datasets used and analyzed during the current study are available from the corresponding author on reasonable request.
