# Supplementary figures and images for: Epidemiology and outcomes of critically ill patients in the emergency department of a tertiary teaching hospital in Rwanda
Source: Int J Emerg Med. 2024 Nov 5;17:170. doi: 10.1186/s12245-024-00736-9 (PMC11536832; doi:10.1186/s12245-024-00736-9)

**Supplemental Figure 1.** modified South African Triage Score (mSATS)


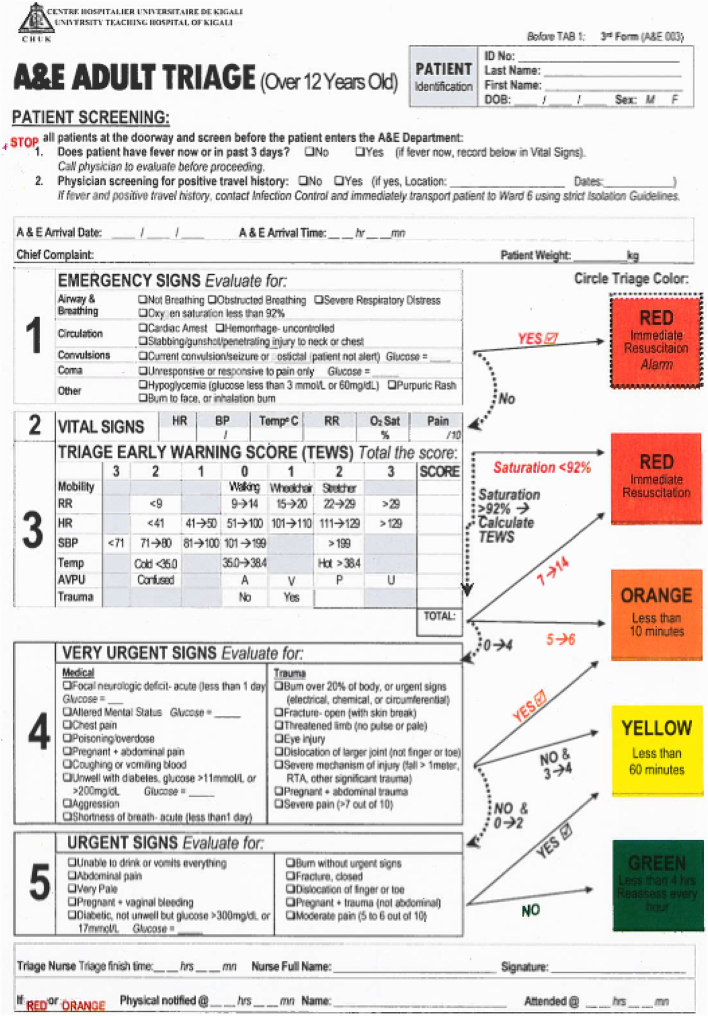

Supplement: Supplementary file 1 — Supplementary Material 1 [file 12245_2024_736_MOESM1_ESM.docx]
